# Questioning assumptions about the abuse potential of medical cannabis and cannabinoids: narrative review and commentary

**DOI:** 10.1192/bjb.2025.10183

**Published:** 2026-08

**Authors:** Peter Pressman, Andrew Wallace Hayes

**Affiliations:** 1 Department of Sociology, The University of Maine, Orono, Maine, USA; 2 College of Public Health, University of South Florida, Tampa, Florida, USA

**Keywords:** Cannabinoids, substance use disorders, psychotic disorders/schizophrenia

## Abstract

**Aims and method:**

There is a pressing need for standardised and comprehensive research frameworks to evaluate both the therapeutic benefits and risks of dependency and misuse associated with medical cannabis. We address the issue of abuse potential or misuse liability by examining research data and what amounts to existing clinical dogma for validity and generalisability. We undertook a broad scoping approach to exploring recent literature, focusing on clinical studies that investigated abuse/misuse and dependence among users of medical and/or recreational cannabis and cannabinoids. The search provided over 350 articles, mostly (∼90%) dated post 2002. Abstract analysis narrowed the selection to under 50 papers, according to their relevance as judged by the authors, an experienced clinician and a public health toxicologist.

**Results:**

We identified and commented upon some broad assumptions within the abuse liability literature that included (a) a standard cannabis formulation; (b) standard routes of administration and standard potency dosing; (c) a standard pattern of use; (d) a standard user or patient; and (e) a standard vulnerability to misuse or dependence.

**Clinical implications:**

Unpacking and questioning these assumptions leads to the conclusion that far more rigorous language and research design are needed to address the question of cannabis abuse definitively but, based on the best available evidence, it appears that the abuse liability of medically supervised cannabis is comparable to any other class of widely used and well-regulated pharmaceutical agents.

The pharmacological investigation of cannabis has a long and complex history, dating back to the early 19th century in Western science, and over thousands of years in traditional medicine. However, modern scientific exploration truly accelerated only after the 1960s, with the isolation of cannabinoids and discovery of the endocannabinoid system. One area that has been shrouded in a scientific and clinical ‘fog of war’ is that of abuse or misuse liability. Determination of the character and magnitude of these risks of medical cannabis is critical for multiple reasons, including public health, regulatory policies and patient safety. By focusing on purpose, regulation and composition, medical cannabis is distinguished as a physician-supervised therapeutic product, whereas recreational and non-medical cannabis are unsupervised and address other needs (Table 1). Key considerations include the following.

- **Public health implications**
  - Misjudging the abuse liability of medical cannabis can lead to increased rates of dependence or misuse among users, particularly in genetically, psychiatrically and socially vulnerable populations.
  - Accurate evaluation of abuse liability in the clinical setting helps in balancing the therapeutic benefits of cannabis with its putative risks. Experience highlights the need for responsible monitoring to avoid unintended consequences of tolerance and overuse.^4^
- **Informed regulatory policies**
  - Regulatory bodies, such as the U.S. Food and Drug Administration (FDA) and U.S. Drug Enforcement Administration, ideally rely on evidence-based abuse liability assessments to classify substances under the Controlled Substances Act. This classification is intended to guide the medical use of, availability of and restrictions on cannabis.^5^
  - Without accurate data, there is a risk of inappropriate scheduling, idiosyncratic prescribing practices and either under- or over-restriction of access.
- **Patient safety and prescriber guidance**
  - Patients using cannabis for medical purposes, particularly those with chronic pain or psychiatric conditions, may be at higher risk of developing substance use disorders if the abuse liability is underestimated.^6^
  - As with any drug, clinicians need clear guidance on safe prescribing practices, indications, product formulations, dosing limits and monitoring protocols to minimise abuse and dependency risks.
- **Prevention of misuse and diversion**
  - Inaccurate assessment of misuse/abuse potential can lead to cannabis being diverted for recreational use, contributing to broader societal issues such as youth access and increased driving under the influence.^4^
  - Accurate evaluations also help implement evidence-based individual and community-based prevention and intervention programmes.

**Table 1:** Comparison between medical and non-medical cannabis Table 1 long description.

Criterion	Medical cannabis	Non-medical cannabis (recreational)
Purpose	To explore therapeutic potential in conditions such as chronic pain, epilepsy or anxiety	Used for recreational enjoyment and psychoactive effects
Cannabinoid profile	Typically tailored cannabinoid ratios (e.g. CBD:THC balance) to suit medical needs; often high-CBD products with lower THC	Often bred for high THC levels to maximise psychoactive effects; lower emphasis on therapeutic balance
Legal requirements	Requires a physician’s recommendation, prescription or enrolment in a medical cannabis programme	Typically available in regions where recreational cannabis is legalised and no medical recommendation is required
Regulation	Heavily regulated for safety, potency and contamination; strict quality assurance protocols	Less stringent in some jurisdictions; focus on potency labelling rather than medical efficacy
Distribution	Dispensed through licensed medical dispensaries or healthcare providers	Sold in retail or recreational dispensaries, often with age restrictions
Labelling	Includes detailed dosing instructions, cannabinoid content and medical warnings	Focuses on strain type, THC percentage and psychoactive effects without medical guidance
Public perception	Increasingly recognised as a legitimate therapeutic tool	May still carry social stigma, depending on cultural and regional attitudes
Side-effects management	Use often monitored by healthcare providers to manage potential side-effects	Use is typically self-monitored, with less guidance on side-effects

THC, tetrahydrocannabinol; CBD, cannabidiole.

Adapted from references.^1–3^

If we can then stipulate that the challenge of evaluating abuse liability is important and requires discrimination between medical and non-medical use and users, the task is to characterise abuse/misuse concerns and their origin, and begin to suggest how and why those concerns differ by type of use and user. Failure to adequately appreciate the divergence between formulations, major cannabinoids and the types of cannabis use may restrict the prescription of a class of ancient compounds whose pharmacologic potentials are only beginning to be explored.

## Method

This Commentary was developed through a narrative review of literature deemed most relevant by the authors to address the provocative topic at hand. The selection process prioritised peer-reviewed articles; primary research and reviews providing foundational evidence; and novel analysis or interpretation. Our search was conducted using PubMed, PsycINFO, Scopus, ISI Web of Science and Google Scholar and employing search terms that included ‘cannabis and abuse or dependence’, ‘cannabinoids and abuse or dependence’, ‘abuse or dependence and medical cannabis or cannabinoids’ and ‘abuse or dependence and recreational cannabis or cannabinoids’. Reviews were excluded except for those presenting novel or foundational analysis or interpretation. The inclusion of sources was guided by their contribution to the Commentary’s objectives rather than by systematic or exhaustive review. Priority was given to work that provided novel insights, corroborated or qualified existing discussion or highlighted gaps in current understanding. While the Commentary does not aim to be a comprehensive review, it seeks to synthesise pivotal views and contextualise them within the broader discourse. The authors acknowledge that the selection of literature reflects subjective judgement, which may have influenced the representation of evidence. However, the sources cited are intended to provide a balanced foundation for the perspectives presented herein and, at least, offer the rationale for more exhaustive and focused study.

## Results

### Cannabis: concerns, origins and threats to the validity of abuse/misuse potential

Cannabis abuse is a term describing the continued pathological use of cannabis. In DSM-5, the American Psychiatric Association defines cannabis use disorder (CUD) in terms of discrete patterns classified under impaired control, social impairment, risky behaviour or physiological adaptation, all with varying degrees of intensity.^7^

Cannabis use disorder is said to affect about 10% of the 193 million cannabis users globally, and it encompasses the possibility that people may suffer adverse consequences because of cannabis use, i.e. beginning with, or leading to, misuse/abuse, without necessarily becoming addicted. Importantly and ironically, the struggle to establish criteria for a continuum of problematic use, abuse or misuse, dependence or addiction seems to contribute more to diagnostic and classificatory confusion rather than clarity.^8^

Cooper and Abrams^9^ have observed that there is relatively little quality research regarding conditions under which cannabis abuse and dependence are more likely to emerge. Schlossarek et al^10^ undertook a literature search of 10 568 studies on the subject. Twenty-six of these finally met the inclusion criteria, and the group’s conclusions included an overarching statement about how striking it was that some studies used terms such as problematic use, abuse and dependence while lacking a precise operationalisation or using the term ‘dependence’ even if the required number of symptoms was not fulfilled. Often, important information on patterns of use, method of use or administration, quantity and quality (type, potency, purity and formulation) was absent, as well as details of context, motives and subjective effects. Importantly, there was little distinction between recreational use and recreational products versus medical use and medical cannabinoids (such as Marinol (dronabinol), the synthetic *Δ* ^9^-tetrahydrocannabinol (*Δ* ^9^-THC) approved in 1985 by the FDA for chemotherapy-associated nausea and anorexia.

Analysis of the strongest studies seemed to yield factors that were associated with an impact on the transition from cannabis use to dependence. Perhaps not surprising was that a wide range of psychiatric disorders was associated with an elevated risk of becoming dependent. The development of a dependence syndrome was also linked with an aetiology in which social, biological and intra-individual factors interact in a complex fashion, leading to dependence. Nevertheless, the putative link between cannabis dependence and predisposing factors was not readily resolved by most studies, due to definitional and methodological weaknesses regarding dependence criteria.

Thus, it is suggested that CUD may more accurately be a proxy for an overarching psychiatric diagnosis that creates psychosocial vulnerabilities for what has been traditionally termed substance abuse. Another important challenge to a broad characterisation of ‘abuse liability’ that lumps every cannabis user into a potential abuse/misuse category lies in genomic variability. Johnson et al^11^ conducted a large-scale, genome-wide association study meta-analysis of cannabis use disorder and found associations with two genome-wide significant loci: a novel chromosome 7 locus (*FOXP2*, lead single-nucleotide polymorphism (SNP) rs7783012) and the previously identified chromosome 8 locus (near *CHRNA2* and *EPHX2*, lead SNP rs4732724). Cannabis use disorder was also positively genetically correlated with other psychopathology, including attention-deficit hyperactivity disorder, major depression and schizophrenia, consistent with the findings of Schlossarek et al.^10^

Gender differences in response to cannabis exposure suggest yet another source of variability. Cooper and Haney^12^ found that, among cannabis smokers, men exhibit greater cannabis-induced analgesia compared with women. Such a gender-dependent difference appears to be independent of the cannabis-elicited psychoactive effects associated with promotion of abuse liability. Thus, gender-dependent differences in cannabis’s analgesic effects appear, at least, to be an important research pathway that justifies further investigation.

### Variability in dose and route of administration

The challenge of dose titration in the medical use of cannabis is complicated by the high degree of variability observed in individual responses to ingested *Δ* ^9^-THC. Clinical experience with dronabinol, a synthetic isoform of *Δ* ^9^-THC, suggests that, for some individuals, 2.5 mg is sufficient to produce apparent effects. In contrast, higher doses are necessary for others – in some cases exceeding 50 mg (reviewed in Grotenhermen^13^). In light of this variability, probably influenced by gender, age and genomics, calculation of pharmacologic potency between a given quantity of *Δ* ^9^-THC contained in smoked cannabis and a mass of *Δ* ^9^-THC contained in an edible product, for example, is extremely difficult.

First-pass metabolism is also a potentially significant source of dose variability; liver enzymes hydroxylate *Δ* ^9^-THC to form 11-hydroxytetrahydrocannabinol (11-OH-THC), a more potent psychoactive metabolite that readily crosses the blood–brain barrier (BBB).^14^ 11-OH-THC is more potent than *Δ* ^9^-THC,^15,16^ and its serum levels are higher when *Δ* ^9^-THC is ingested orally than when it is inhaled.^13^ In addition to inhaled (smoked, vaporised or aerosolised) and oral administration, administration of medical cannabinoids via other modes is being explored. Oral-mucosal, sublingual, transdermal, transrectal and transvaginal formulations are under preclinical investigation. Each mode of drug delivery has its own spectrum of complexity and, as yet, incomplete characterization of its absorption/distribution/metabolism/excretion, and it will be interesting to evaluate these data as they emerge.

### Withdrawal

Withdrawal is an essential component of classical addiction theory and is a major criterion used to determine whether an agent is addictive or subject to recurrent abuse or misuse liability; it is a vital manifestation of dependence.^17^ The physical dependence on substances of abuse/misuse is phenomenologically likely to involve repeated cycles of withdrawal accompanied by increased motivation to self-administer that substance. This phenomenon is generally not seen with medical cannabis, and is unusual in the realm of recreational cannabis use. There is longstanding evidence, however, of a withdrawal syndrome reliably following abrupt discontinuation of chronic heavy cannabis use.^18^

Withdrawal symptoms in patients with neuropathic pain treated with the cannabinoid dronabinol or *Δ* ^9^-tetrahydrocannabinol, a synthetic form of tetrahydrocannabinol (THC), included only mild and transient sleep disturbance, anxiety and increase in neuropathic pain.^19^

The Schimrigk trial was designed to explore the positive benefit/risk ratio of dronabinol among 240 multiple sclerosis patients with central neuropathic pain. The trial protocol was a 16-week, placebo-controlled Phase III study followed by a 32-week, open-label period. One hundred patients received therapy for up to 119 weeks. The primary end-point was a change in pain intensity throughout the treatment period. Safety evaluation was based on sequelae of dependency, and abuse. No signs of drug abuse, and only one possible case of dependency, were reported. Consistent with decades of clinical experience with Marinol or dronabinol, the trial suggests safety over a significant duration.

For most patients, no withdrawal reactions were reported following cessation of study medication: withdrawal symptoms were reported for six patients following the open-label period and for four patients after the extended follow-up. Importantly, diagnostic criteria for drug dependency and abuse were serially assessed by investigators during the long-term follow-up period. Mild signs of drug dependency were reported for one patient.

In another older study, for clinical trials of Sativex (a THC + cannabidiole (CBD) medicine), intoxication scores were low and euphoria was reported by only 2.2% of subjects. Tolerance did not occur, abrupt withdrawal failed to show a stereotypic withdrawal syndrome and no cases of abuse were reported in follow-up. A formal abuse liability study of Sativex in long-term cannabis smokers suggested some abuse potential in comparison with a placebo.^20^

### Synthetic cannabinoids

It has been emphasised that much of our albeit flawed understanding of cannabinoid tolerance, dependence and withdrawal has been based on experience with *Δ* ^9^-THC, a relatively weak partial agonist at CB1 and CB2 receptors.^21^ However, the synthetic cannabinoids (SCBs) commonly found in commerce and on the ‘black market’, such as K2 and Spice, are typically full cannabinoid receptor agonists. The authors point out that a low-efficacy cannabinoid like *Δ*9-THC will have a less pronounced maximal effect than higher-efficacy cannabinoids, such as the SCBs, and this difference in maximal effects cannot be overcome simply by increasing the dose of *Δ* ^9^-THC. In other words, no amount of *Δ* ^9^-THC can stimulate cannabinoid receptors to the same degree as the SCBs currently emerging as a principal subclass of cannabinoid drugs of abuse. This situation has left investigators working with efficacy SCBs in the difficult position of having to tease out whether the effects are related to the significant degree of cannabinoid receptor stimulation produced by these compounds, or whether interactions with other non-cannabinoid receptors or even non-receptor systems produce them. Thus, there is greater confusion in the increasingly dense ‘fog of war’ in understanding cannabinoid use and possible abuse/misuse.

### Cannabis as a ‘gateway’ drug

The question of cannabis as a gateway drug is integral to the discussion of the linkage of this substance to other established drugs of abuse/misuse. Any gateway effect is more likely to be driven by social, psychological and biological vulnerabilities, not by the pharmacological properties of cannabis itself. There is no proven causal link between cannabis use for medical indications and progression to more potent or dangerous agents,^22^ and medical cannabis often substitutes for opioids and other medications.^23,24^

A report prepared by the Federal Research Division, Library of Congress, under an Interagency Agreement with the Office of the Director, National Institute of Justice, Office of Justice Programs, U.S. Department of Justice in November 2018 (https://www.ojp.gov/pdffiles1/nij/252950.pdf), stated:

‘The existing statistical research and analysis show mixed results and do not clearly demonstrate scientific support for cannabis use, leading to harder illicit drug use. As a result, FRD has determined that no causal link between cannabis use and the use of other illegal drugs can be claimed at this time.’

The report goes on to cite deficiencies or weaknesses in the reviewed research. It is noted that many studies of cannabis use are based on self-reported data from longitudinal or retrospective studies that suffer from bias; it seems axiomatic at this point that subjects often cannot accurately recall the specifics requested, tend to provide responses that they believe investigators want to hear or even portray themselves in a favourable light.

Other studies may collect biased data by sampling from heroin users, street youth and other at-risk populations. As such, the results are not readily generalisable. The limitations of animal models are also noted, along with the constraints of translating findings to human behaviour.

Finally, it is noted that 2018 statistics from the U.S. Department of Health and Human Services Substance Abuse and Mental Health Services Administration show that only 0.3% of those Americans who have used cannabis have abused heroin, 0.2% have abused cocaine and 0.1% have abused methamphetamines.

It also should be noted that the findings from another frequently cited study are consistent with those of the Federal Research Division report: it was found that the initiation of cannabinoid-based medicines in patients with chronic pain was associated with 17-fold higher odds of ceasing prescription analgesics within 21 months.^25^

On the other hand, one frequently cited study^26^ reported that 44.7% of individuals with lifetime cannabis use progressed to other illicit drug use at some time in their life. The fact remained, however, that several antecedent variables, the most important being underlying psychiatric disorder, were found to predict progression from cannabis use to other illicit drugs use.

### Dosing mechanisms

Dose-response patterns in cannabinoids appear to be hormetic or biphasic, i.e. at lower doses there appear to be salutary effects whereas higher dose ranges are associated with undesirable and potentially hazardous outcomes. The mechanisms involved may also be either receptor- or non-receptor-based.

The consistent quantitative features of this biphasic dose response at the cellular level may be complicated by the effects of upstream and highly conserved gene clusters that control and direct the allocation of the metabolic programming of the entire organism.^27,28^

Current evidence suggests that THC is anxiolytic at low doses and anxiogenic at higher doses, with each case demonstrating unique dose-response curves. Interestingly, CBD (possibly in conjunction with other cannabis plant constituents such as cannabinol, terpenes and flavonoids) may help to lower or antagonise the psychotropic effects of THC when used in particular combinations and proportions, an observation that underscores the priority for understanding the interaction between these two cannabinoids.^29,30^

Analyses of the overall safety of cannabinoid-based medicines are further confounded by variations in dosing and administration methods across products, study designs and indications. While there are standardised doses for CBD use in Lennox-Gastaut and Dravet syndromes, and in investigational studies, a commonly used approach to dosing is ‘start low, go slow, stay low’.^31,32^ While this ‘personalised’ or ‘precision’ medicine approach is helpful for patients as individuals, lack of standardisation, even within the same indication, represents a challenge for the overall analysis of safety, and further studies to establish appropriate standard doses or dose ranges for each indication are needed. Additionally, while many cannabinoid-based medicines are administered orally, inhalation – and, in particular, vaporisation, not smoking – may be deemed most appropriate. Table 2 summarises research on abuse potential that counters many of the conclusions and assumptions suggesting elevated abuse potential.

**Table 2:** Abuse characteristics of medical cannabis Table 2 long description.

Evidence source	Study design and population	Key evidence	Abuse potential conclusion
Systematic Review & Meta-analysis^33^	Reviews of RCTs in chronic pain, spasticity and chemotherapy-induced nausea	RCTs generally show modest improvements in symptoms; adverse events (e.g. dizziness, sedation) are common, but withdrawal due to adverse events is infrequent	No consistent evidence that therapeutic use increases abuse probability
Cochrane Review^34^	RCTs of medical cannabinoids for pain and spasticity (predominantly patients with neuropathic pain)	Benefits are small, and effects on pain are borderline; adverse events are common but rarely lead to treatment discontinuation	Limited evidence of abuse liability in controlled medical settings
Observational Data & Guidelines^35^	Studies in medical settings with controlled dosing and patient selection	Observational studies report that most patients using medical cannabinoids are experienced cannabis users; rates of cannabis use disorder are similar in medical and recreational populations	No clear signal that medical use (with proper screening and dosing) increases the risk of abuse compared with background rates
Reviews on Cannabinoid Dependence^36^	Summaries of epidemiologic data on cannabis use disorder across various populations	Lifetime risk of cannabis dependence among users is estimated at approximately 9%; in medical settings, careful patient selection limits risk	Abuse potential appears inherent to cannabis but is not exacerbated by medical use per se

RCT, randomised controlled trial.

### Cannabis itself

There is little standardisation, consistency or harmonisation in commerce in regard to cannabis. This plant can provide over 500 different active chemical compounds that interact with numerous molecular targets across several receptor families, thereby modulating the production and metabolism of endocannabinoids, gamma-aminobutyric acid, glutamate and serotonin. Unlike 24 synthetics and alcohol, cannabis is a more complex drug(s). Consumption or inhalation of the botanical resin exposes the user to hundreds of compounds, including both cannabinoids (e.g. THC and cannabidiol) and non-cannabinoids (i.e. terpenes and flavonoids), many of which are bioactives. This is in direct contrast to isolated pharmaceutical derivatives (e.g. dronabinol and cannabidiol). The sheer complexity of the plant makes comparison difficult even between THC and CBD.^37^

Psychoactive effects are primarily derived from THC, which is a partial agonist at both cannabinoid CB1 and CB2 receptors. CB1 receptors are located throughout the central nervous system, lungs, liver and kidneys. Binding CB2 receptors modulates G-protein-coupled inhibition of cyclic adenosine monophosphate (cAMP), thereby influencing pain, mood, appetite and sexual activity. Interaction between exogenous cannabinoids and endocannabinoid tone is another fertile area for augmenting the understanding of mechanisms of action and the array of salutary and adverse effects.

It has been pointed out that the two most studied exogenous cannabinoids are THC and CBD.^38^ CBD does not appear to bind with significant affinity to CB1 or CB2 receptors but seems to possess independently modulated neuroprotective and anti-inflammatory effects. Several potential CBD actions have been proposed: inhibition of cyclooxygenase and lipoxygenase, inverse agonism at CB1/CB2 receptors and enhancement of anandamide (an endogenous THC analogue). It is proposed that CBD may be effective in epilepsy through modulation of the endocannabinoid system by halting the degradation of anandamide, which may have a role in inhibition of seizures. Additionally, CBD may play a role in regulation of T-type calcium channels and nuclear peroxisome proliferator-activated receptor-γ, both of which have been implicated in seizure activity.

### Metabolites of cannabis

Hepatic metabolism can produce over 80 metabolites of *Δ* ^9^-THC, with the most common pathway involving allylic hydroxylation at the 11-position followed by oxidation to a carboxy derivative. Conjugation occurs with some metabolites, but it does not appear to be a major step. Bioavailability varies depending on the user’s smoking topography, such as number, duration, spacing of puffs, hold time and inhalation volume. It has long been known that THC remains in the body for extended periods due to its lipophilic properties, allowing it to accumulate and be released slowly from adipose tissue.^15^ It is tempting to speculate about delayed and unintended effects in those engaging in rapid weight loss.

It cannot be stressed too much that some of the differences and confounds between the properties of recreational versus medical cannabis are exemplified by the frequency of what appears to be sloppy comparison across various agents. Dronabinol, for example, contains only the synthetic version of *Δ* ^9^-THC whereas cannabis products may contain *Δ* ^9^-THC plus an array of cannabinoids and other compounds, including terpenes and cannaflavins.^39^

### Variability in manufacture

Because of non-standard extraction and formulation, the amount of *Δ* ^9^-THC in edibles can vary across a single product type, and across batches processed at different times (brief shelf-life), making it challenging for users to estimate how much *Δ* ^9^-THC they consume. Apart from accessibility and the visual appeal of packaging, this fact may explain in part why some edible products are also associated with cannabis-related childhood poisoning.

Lower THC in the plasma may be the result of generally low bioavailability: although in a more potent form, the amount of THC that reaches the circulation following oral administration may be only be 6–10% of the amount contained in the product.^40^ The lack of product consistency, together with delayed intoxication (attributed to the time required for gastrointestinal absorption, possible food matrix and first-pass metabolism), may cause users of cannabis to consume higher than intended amounts of the drug. Edible products such as gummies are responsible for the majority of healthcare visits due to cannabis intoxication, which is often due to ignorance and inexperience in naïve users or their failure to appreciate the delayed effects.^41^

### Variability in self-dosing

Dosage estimation for existing retail products is often inaccurate.^41^ While state laws in the USA often require that total milligrams of *Δ* ^9^-THC and the number of servings be included on packages available for retail sale, doses are likely to vary widely. Individuals often tell a story about having eaten the suggested serving size initially, followed by a decision to consume the entire edible product after not feeling any effects. They also report that it was both easy and likely that they would consume the entire edible product in one sitting, just as they would a normal baked good,^39^ suggesting at least the likelihood of, or vulnerability to, a persistent pattern of misuse.

Gottschling et al^42^ provide additional emphasis that recreational cannabis use may be associated with uncertain product composition, with potentially high THC concentrations and unknown biologically active impurities. Again, dosing may be unknown and uncontrolled and usage may be accompanied by concomitant tobacco or other recreational drug use, each with its own set of independent and potential interactive effects.

### Discussion

#### The present

The existing risk assessment literature on cannabis use appears to be rife with conflicting findings. Much research seems to be based on one or more assumptions that are simplistic at best and, at worst, simply unsupported. The dual narratives of cannabis safety reflect the lack of a standardised scientific and clinical environment that supports the safe integration of cannabinoids into both mainstream medical and recreational practice. The absence of standardisation is a theme that runs through virtually every level of analysis when the question of safety is raised; there is no ‘standard’ cannabinoid preparation but there is a multitude of cannabinoids, formulations, potencies and routes of administration. Manifestations of this theme include the following: cultivars and species vary widely – cannabis contains well over 100 phytocannabinoids, and most pharmacological effects are unknown. Of those that are known, their extraction and processing vary widely. Drug delivery systems vary widely, and some are adulterated by users to increase exposure and/or potency.

There is poor standardisation and wide variety in definitions of the drug or product itself, potency, route of administration, abuse, misuse, dependence and withdrawal. DSM-V criteria for cannabis-related disorders are therefore arguably inadequate. Available tools to diagnose and detect substance use disorders designed for recreational drug use may then not be suitable for evaluating and characterising substance misuse in patients. The DSM-5 criteria for cannabis-related disorders, then, do not account for the diversity of cannabis products and uses; pathologise tolerance and withdrawal even in legitimate medical use; rely on arguably ambiguous and context-insensitive criteria; use diagnostic tools designed for illicit or recreational users; and lack standardisation around dose, potency and method of use. This constellation of qualifications may be seen as leading to misclassification of medical users as disordered, inappropriate treatment interventions, and to stigmatisation of therapeutic cannabis use. There appears to be a growing consensus among clinicians that new, tailored diagnostic frameworks are needed for cannabis – especially in the evolving landscape of legal medical and recreational use.

There are few standardised ‘good manufacturing practices’ across the cannabis industry. Studies in healthy recreational users versus medically ill patients versus surgical patients versus chronic pain patients yield very different results; an amalgamation of disparate data has led to confusion around the safety profiles of cannabinoid-based medicines. Studies of recreational cannabis use are associated with several confounding factors that may not apply to cannabinoid-based medicines.

Recreational cannabis use may be associated with uncertain product compositions, with potentially a very high content of THC (or synthesised equivalent) and unknown impurities. Moreover, dosing may be unknown and uncontrolled, and usage may be accompanied by concomitant tobacco or other recreational or prescription drug use. More studies on specific indications are needed to establish the frequency, intensity and duration of adverse events, and to optimise dosing for relevant patient populations and sub-populations.

There is a need for academic and industry partnerships to achieve standardised, more comprehensive and rigorous research on cannabis and cannabinoids in general, and on the development of safe and effective cannabis-driven products.

Based on the best available evidence, the abuse liability of medically supervised cannabis is comparable to any other class of pharmaceutical agents. We do need, however, more accurate terminology and more rigorously designed studies to definitively address the question of cannabis abuse potential.

#### The future

It has been observed that, in the recreational and medical realms, cannabis use is increasing and moving faster than the associated science (https://www.fundacion-canna.es/en/meet-experts-interview-ziva-cooper). This description may be the best summary of the state of cannabis research at this time. Apart from the fusion and confusion of weak and strong data in the current body of associated research, a bright future is predicted: cannabis will be vaporised, rather than smoked, which is probably safer and more effective for therapeutic purposes; and it will be examined in an array of forms, doses, combinations and formulations for a growing number of clinical indications. (Importantly, inhalational risks of any inhalational aerosol may include vitamin E acetate in various diluents, propylene glycol and heavy metals, any or all of which may be associated with airway irritation, inflammation and even pneumonia, and the unpredictability of onset and dose.)

The idea of the entourage effect, that a combination of terpenes, flavonoids and cannabinoids is more beneficial than cannabinoid isolates, is important to examine. There is, of course, a need for well-designed randomised, double-blind, placebo-controlled studies of adequate size and duration – especially in the promising area of analgesia and augmentation of analgesia affected by other (opiate) agents.

Other work on novel synthetic cannabinoid ligands, such as KM-233, is intriguing; this is a classic cannabinoid with a BBB penetration attribute and possessing a selective affinity for CB2 receptors. Like *Δ* ^8^-THC, KM-233 shares the tricyclic core structure of cannabinoids but has been chemically modified to enhance CB2 receptor selectivity, BBB penetration and anti-tumour properties. KM-233 has shown cytotoxic effects on U87 cells, probably via CB2 receptor-mediated apoptosis, inhibition of tumour cell proliferation and modulation of immune response within the tumour microenvironment. *Δ* ^8^-THC itself has certain cytotoxic effects on glioma cells but is less potent and less selective compared with KM-233. KM-233 is seen as promising in regard to its cytotoxicity against human U87 glioma/glioblastoma cells.^43^

Ultimately it may be that the most exciting breakthrough in terms of impact in the near term, both in research and clinical indication, lies with the biosynthesis of pure, standardised cannabinoids produced in a cost-effective fashion using gene editing and fermentation technology, i.e. to have yeast produce safe, abundant and inexpensive cannabinoids.^44^

#### Improving safety

Risks, especially in the recreational realm, might be reduced through standardisation of product formulations, adequate quality control measures and appropriate product labelling – trends that seem to be increasingly embraced throughout the growing industry. On the extraction/processing/production/analysis side, much remains to be proven, standardised and implemented to ensure consistent dosage and a uniform product. On the labelling side, more should be done to ensure that consumers are better educated on how cannabinoids may affect the body, and that they become aware of how to use cannabis safely to avoid concerns such as unintentional intoxication or aversive ‘highs’ lasting longer than anticipated.

#### Limitations

This Commentary reflects the perspective and experiences of the authors, which may introduce inherent biases. It is shaped by subjective interpretations and clinical experience which, while valuable, may not comprehensively represent the full spectrum of viewpoints or empirical evidence on the topic. The absence of systematic methodologies or external validation may limit the generalisability of the insights and opinions presented. A 2022 commentary by Eisenberg described current cannabis research as a ‘Bermuda Triangle of low quality studies, countless meta-analyses and conflicting recommendations’.^45^ Readers are encouraged to consider the present Commentary as one of multiple evolving perspectives, and to conduct and consult well-designed and robust studies for a balanced understanding of the subject matter.

It is important to note that some cannabis-related issues in the UK differ from those in North America and much of Europe, reflecting unique legal, medical and regulatory dynamics. Although medical cannabis has been legal in the UK since 2018, access remains limited, with National Health Service prescriptions rare and most patients reliant on private clinics. Prescribing is restricted to specialists, and caution within the medical community has surely constrained adoption. The UK’s illicit cannabis market appears to be dominated by high-THC, low-CBD strains (often referred to as ‘skunk’), which have been linked in UK-specific studies to increased risks of psychosis – shaping a public discourse heavily focused on mental health harms. Paradoxically, the UK is a leading global exporter of medical cannabis. Meanwhile CBD regulation remains stringent, with novel food classifications complicating retail access. Cannabis policy reform seems to remain an uphill political battle. Together, these factors create a uniquely cautious landscape for cannabis in the UK, distinct from models seen elsewhere.

## Data Availability

Data availability is not applicable to this article because no new data were created or analysed.

## About the authors

**Peter Pressman**, MD, works in the Department of Sociology, University of Maine, Orono, Maine, USA. **Andrew Wallace Hayes**, PhD, works at the College of Public Health, University of South Florida, Tampa, Florida USA.

## Table 1 Long description

A table comparing medical cannabis and non-medical cannabis across various criteria. The table has 8 rows and 3 columns. The columns are labeled Criterion, Medical cannabis, and Non-medical cannabis (recreational). Row 1: Criterion, Medical cannabis, Non-medical cannabis (recreational). Row 2: Purpose, To explore therapeutic potential in conditions such as chronic pain, epilepsy or anxiety, Used for recreational enjoyment and psychoactive effects. Row 3: Cannabinoid profile, Typically tailored cannabinoid ratios (e.g. CBD:THC balance) to suit medical needs; often high-CBD products with lower THC, Often bred for high THC levels to maximise psychoactive effects; lower emphasis on therapeutic balance. Row 4: Legal requirements, Requires a physician's recommendation, prescription or enrolment in a medical cannabis programme, Typically available in regions where recreational cannabis is legalised and no medical recommendation is required. Row 5: Regulation, Heavily regulated for safety, potency and contamination; strict quality assurance protocols, Less stringent in some jurisdictions; focus on potency labelling rather than medical efficacy. Row 6: Distribution, Dispensed through licensed medical dispensaries or healthcare providers, Sold in retail or recreational dispensaries, often with age restrictions. Row 7: Labelling, Includes detailed dosing instructions, cannabinoid content and medical warnings, Focuses on strain type, THC percentage and psychoactive effects without medical guidance. Row 8: Public perception, Increasingly recognised as a legitimate therapeutic tool, May still carry social stigma, depending on cultural and regional attitudes. Row 9: Side-effects management, Use often monitored by healthcare providers to manage potential side-effects, Use is typically self-monitored, with less guidance on side-effects.

Navigate back to Table 1.

## Table 2 Long description

A table summarizing research on abuse potential of medical cannabis. The table has four rows and four columns. The columns are labeled 'Evidence source', 'Study design and population', 'Key evidence', and 'Abuse potential conclusion'. Row 1: Evidence source, Systematic Review & Meta-analysis; Study design and population, Reviews of RCTs in chronic pain, spasticity and chemotherapy-induced nausea; Key evidence, RCTs generally show modest improvements in symptoms; adverse events (e.g. dizziness, sedation) are common, but withdrawal due to adverse events is infrequent; Abuse potential conclusion, No consistent evidence that therapeutic use increases abuse probability. Row 2: Evidence source, Cochrane Review; Study design and population, RCTs of medical cannabinoids for pain and spasticity (predominantly patients with neuropathic pain); Key evidence, Benefits are small, and effects on pain are borderline; adverse events are common but rarely lead to treatment discontinuation; Abuse potential conclusion, Limited evidence of abuse liability in controlled medical settings. Row 3: Evidence source, Observational Data & Guidelines; Study design and population, Studies in medical settings with controlled dosing and patient selection; Key evidence, Observational studies report that most patients using medical cannabinoids are experienced cannabis users; rates of cannabis use disorder are similar in medical and recreational populations; Abuse potential conclusion, No clear signal that medical use (with proper screening and dosing) increases the risk of abuse compared with background rates. Row 4: Evidence source, Reviews on Cannabinoid Dependence; Study design and population, Summaries of epidemiologic data on cannabis use disorder across various populations; Key evidence, Lifetime risk of cannabis dependence among users is estimated at approximately 9 percent; in medical settings, careful patient selection limits risk; Abuse potential conclusion, Abuse potential appears inherent to cannabis but is not exacerbated by medical use per se.

Navigate back to Table 2.

## References

[1] National Academies of Sciences, Engineering, and Medicine. The Health Effects of Cannabis and Cannabinoids: The Current State of Evidence and Recommendations for Research. The National Academies Press, 2017. Available from: 10.17226/24625. 28182367

[2] National Institute on Drug Abuse. Marijuana as Medicine. National Institute on Drug Abuse, 2020.

[3] World Health Organization. Cannabidiol (CBD) Critical Review Report: Expert Committee on Drug Dependence Fortieth Meeting Geneva, 4–7 June 2018. GRECC, 2022. Available from: https://www.grecc.org/publications/dossiers-scientifiques/cannabidiol-cbd-critical-review-report-who-2018/.

[4] Volkow ND , Baler RD , Compton WM , Weiss SRB. Adverse health effects of marijuana use. N Engl J Med 2014; 370: 2219–27. 24897085 10.1056/NEJMra1402309 PMC4827335

[5] National Institute on Drug Abuse. Is Marijuana Addictive? National Institute on Drug Abuse, 2020.

[6] Hall W , Degenhardt L. Adverse health effects of non-medical cannabis use. Lancet 2009; 374: 1383–91. 19837255 10.1016/S0140-6736(09)61037-0

[7] Patel J , Marwaha R. Cannabis use disorder. In StatPearls. StatPearls Publishing, 2022 (https://www.ncbi.nlm.nih.gov/books/NBK538131/).

[8] Connor J , Stjepanovic D , Le Foll B , Hoch E , Budney A , Hall W. Cannabis use and cannabis use disorder. Nat Rev Dis Primers 2021; 7: 16. 33627670 10.1038/s41572-021-00247-4 PMC8655458

[9] Cooper ZD , Abrams DI. Considering abuse liability and neurocognitive effects of cannabis and cannabis-derived products when assessing analgesic efficacy: a comprehensive review of randomized-controlled studies. Am J Drug Alcohol Abuse 2019; 45: 580–95. 31687845 10.1080/00952990.2019.1669628 PMC7279709

[10] Schlossarek S , Kempkensteffen J , Reimer J , Verthein U. Dependence: a systematic review of the literature. Eur Addict Res 2016; 22: 131–44. 26551358 10.1159/000441777

[11] Johnson EC , Demontis D , Thorgeirsson TE , Walters RK , Polimanti R , Hatoum AS , et al. A large-scale genome-wide association study meta-analysis of cannabis use disorder. Lancet Psychiatry 2020; 7: 1032–45. Erratum in: *Lancet Psychiatry* 2022; 9: e12. 33096046 10.1016/S2215-0366(20)30339-4 PMC7674631

[12] Cooper ZD , Haney M. Sex-dependent effects of cannabis-induced analgesia. Drug Alcohol Depend 2016; 167: 112–20. 27522535 10.1016/j.drugalcdep.2016.08.001 PMC5037015

[13] Grotenhermen F. Harm reduction associated with inhalation and oral administration of cannabis and THC. J Cannabis Ther 2001; 1: 133–52.

[14] Mura P , Kintz P , Dumestre V , Raul S , Hauet T. THC can be detected in brain while absent in blood. J Analyt Toxicol 2005; 29: 842–3. 16356342 10.1093/jat/29.8.842

[15] Hollister LE , Gillespie HK , Ohlsson A , Lindgren J-E , Wahlen A , Agurell S. Do plasma concentrations of delta 9-tetrahydrocannabinol reflect the degree of intoxication? J Clin Pharmacol 1981; 21: 171–7S. 10.1002/j.1552-4604.1981.tb02593.x 6271822

[16] Huestis MA , Henningfield JE , Cone EJ. Blood cannabinoids. I. Absorption of THC and formation of 11-OH-THC and THCCOOH during and after smoking marijuana. J Analyt Toxicol 1992; 16: 276–82. 1338215 10.1093/jat/16.5.276

[17] Piper ME. Withdrawal: expanding a key addiction construct. Nicotine Tob Res 2015; 17: 1405–15. 25744958 10.1093/ntr/ntv048 PMC4654762

[18] Budney AJ , Hughes JR , Moore BA , Vandrey R. Review of the validity and significance of cannabis withdrawal syndrome. Am J Psychiatry 2004; 161: 1967–77. 15514394 10.1176/appi.ajp.161.11.1967

[19] Schimrigk S , Marziniak M , Neubauer C , Kugler EM , Werner G , Abramov-Sommariva D. Dronabinol is a safe long-term treatment option for neuropathic pain patients. Eur Neurol 2017; 78: 320–9. 29073592 10.1159/000481089 PMC5804828

[20] Robson P. Abuse potential and psychoactive effects of δ-9-tetrahydrocannabinol and cannabidiol oromucosal spray (Sativex), a new cannabinoid medicine. Expert Opin Drug Saf 2011; 10: 675–85. 21542664 10.1517/14740338.2011.575778

[21] Tai S , Fantegrossi WF. Synthetic cannabinoids: pharmacology, behavioral effects, and abuse potential. Curr Addict Rep 2014; 1: 129–36. 26413452 10.1007/s40429-014-0014-y PMC4582439

[22] Noel W , Wang J. Is Cannabis a Gateway Drug? Key Findings and Literature Review (NCJ 252950). U.S. Department of Justice, 2018.

[23] Boehnke KF , Scott JR , Martel MO , Smith T , Bergmans RS , Kruger DJ , et al. Substituting medical cannabis for medications among patients with rheumatic conditions in the United States and Canada. ACR Open Rheumatol 2024; 6: 826–35. 39236308 10.1002/acr2.11717 PMC11638128

[24] Reiman A , Welty M , Solomon P. Cannabis as a substitute for opioid-based pain medication: patient self-report. Cannabis Cannabinoid Res 2017; 2: 160–6. 28861516 10.1089/can.2017.0012 PMC5569620

[25] Vigil JM , Stith SS , Adams IM , Reeve AP. Associations between medical cannabis and prescription opioid use in chronic pain patients: a preliminary cohort study. PLOS One 2017; 12: e0187795. 29145417 10.1371/journal.pone.0187795 PMC5690609

[26] Secades-Villa R , Garcia-Rodríguez O , Jin CJ , Wang S , Blanco C. Probability and predictors of the cannabis gateway effect: a national study. Int J Drug Policy 2015; 26: 135–42. 25168081 10.1016/j.drugpo.2014.07.011 PMC4291295

[27] Calabrese EJ. Hormesis: a revolution in toxicology, risk assessment and medicine. EMBO Rep 2004; 5(suppl 1): S37–40. 15459733 10.1038/sj.embor.7400222 PMC1299203

[28] Calabrese EJ , Mattson MP. How does hormesis impact biology, toxicology, and medicine? NPJ Aging Mech Dis 2017; 3: 13. 28944077 10.1038/s41514-017-0013-z PMC5601424

[29] Schoedel KA , Szeto I , Setnik B , Sellers EM , Levy-Cooperman N , Mills C , et al. Abuse potential assessment of cannabidiol (CBD) in recreational polydrug users: a randomized, double-blind, controlled trial. Epilepsy Behav 2018; 88: 162–71. 30286443 10.1016/j.yebeh.2018.07.027

[30] Stewart C , Fong Y. Perioperative cannabis as a potential solution for reducing opioid and benzodiazepine dependence. JAMA Surg 2021; 156: 181–90. 33263719 10.1001/jamasurg.2020.5545

[31] MacCallum CA , Russo EB. Practical considerations in medical cannabis administration and dosing. Eur J Intern Med 2018; 49: 12–19. 29307505 10.1016/j.ejim.2018.01.004

[32] Schuck RN , Pacanowski M , Kim S , Madabushi R , Zineh I. Use of titration as a therapeutic individualization strategy: an analysis of Food and Drug Administration-approved drugs. Clin Transl Sci 2019; 12: 236–9. 30791226 10.1111/cts.12626 PMC6510374

[33] Allan GM , Finley CR , Ton J , Perry D , Ramji J , Crawford K , et al. Systematic review of systematic reviews for medical cannabinoids: pain, nausea and vomiting, spasticity, and harms. Can Fam Physician 2018; 64: e78–94. 29449262 PMC5964405

[34] Whiting PF , Wolff RF , Deshpande S , Di Nisio M , Duffy S , Hernandez AV , et al. Cannabinoids for medical use: a systematic review and meta-analysis. JAMA 2015; 313: 2456–73. 26103030 10.1001/jama.2015.6358

[35] Allan GM , Ramji J , Perry D , Ton J , Beahm NP , Crisp N , et al. Simplified guideline for prescribing medical cannabinoids in primary care. Can Fam Physician 2018; 64: 111–20. 29449241 PMC5964385

[36] National Academies of Sciences, Engineering, and Medicine, Health and Medicine Division, Board on Population Health and Public Health Practice, Committee on the Health Effects of Marijuana: An Evidence Review and Research Agenda. The Health Effects of Cannabis and Cannabinoids: The Current State of Evidence and Recommendations for Research. The National Academies Press, 2017. Available from: https://www.ncbi.nlm.nih.gov/books/NBK423845/. 28182367

[37] Radwan MM , ElSohly MA , El-Alfy AT , Ahmed SA , Slade D , Husni AS , et al. Isolation and pharmacological evaluation of minor cannabinoids from high-potency *Cannabis sativa* . J Nat Prod 2015; 78: 1271–6. 26000707 10.1021/acs.jnatprod.5b00065 PMC4880513

[38] Campbell CT , Phillips MS , Manasco K. Cannabinoids in pediatrics. J Pediatr Pharmacol Ther 2017; 22: 176–85. 28638299 10.5863/1551-6776-22.3.176 PMC5473390

[39] Russo EB. Taming THC: potential cannabis synergy and phytocannabinoid-terpenoid entourage effects. Br J Pharmacol 2011; 163: 1344–64. 21749363 10.1111/j.1476-5381.2011.01238.x PMC3165946

[40] Schwilke EW , Schwope DM , Karschner EL , Lowe RH , Darwin WD , Kelly DL , et al. Delta9-tetrahydrocannabinol (THC), 11-hydroxy-THC, and 11-nor-9-carboxy-THC plasma pharmacokinetics during and after continuous high-dose oral THC. Clin Chem 2009; 55: 2180–9. 19833841 10.1373/clinchem.2008.122119 PMC3196989

[41] Vandrey R , Raber JC , Raber ME , Douglass B , Miller C , Bonn-Miller MO. Cannabinoid dose and label accuracy in edible medical cannabis products. JAMA 2015; 313: 2491–3. 26103034 10.1001/jama.2015.6613

[42] Gottschling S , Ayonrinde O , Bhaskar A , Blockman M , D’Agnone O , Schecter D , et al. Safety considerations in cannabinoid-based medicine. Int J Gen Med 2020; 13: 1317–33. 33299341 10.2147/IJGM.S275049 PMC7720894

[43] Gurley SN , Abidi AH , Allison P , Guan P , Duntsch C , Robertson JH , et al. Mechanism of anti-glioma activity and in vivo efficacy of the cannabinoid ligand KM-233. J Neurooncol 2012; 110: 163–77. 22875710 10.1007/s11060-012-0958-5

[44] Luo X , Reiter MA , d’Espaux L , Wong J , Denby CM , Lechner A , et al. Complete biosynthesis of cannabinoids and their unnatural analogues in yeast. Nature 2019; 567: 123–6. 30814733 10.1038/s41586-019-0978-9

[45] Eisenberg E , Morlion B , Brill S , Häuser W. Medicinal cannabis for chronic pain: the Bermuda Triangle of low-quality studies, countless meta-analyses and conflicting recommendations. Eur J Pain 2022; 26: 1183–5. 35363933 10.1002/ejp.1946 PMC9323487

